# Comparison of Clinical Outcomes and Safety Associated With Chlorthalidone vs Hydrochlorothiazide in Older Adults With Varying Levels of Kidney Function

**DOI:** 10.1001/jamanetworkopen.2021.23365

**Published:** 2021-09-15

**Authors:** Cedric Edwards, Gregory L. Hundemer, William Petrcich, Mark Canney, Greg Knoll, Kevin Burns, Ann Bugeja, Manish M. Sood

**Affiliations:** 1 Ottawa Hospital Research Institute, Division of Nephrology, Department of Medicine, University of Ottawa, Ottawa, Canada; 2 Institute for Clinical Evaluative Sciences, Ottawa, Canada

## Abstract

**Importance:**

Thiazide diuretics are commonly prescribed for the treatment of hypertension, a disease highly prevalent among older individuals and in those with chronic kidney disease. How specific thiazide diuretics compare in regard to safety and clinical outcomes in these populations remains unknown.

**Objective:**

To compare safety and clinical outcomes associated with chlorthalidone or hydrochlorothiazide use among older adults with varying levels of kidney function.

**Design, Setting, and Participants:**

This population-based retrospective cohort study was conducted in Ontario, Canada, from 2007 to 2015. Participants included adults aged 66 years or older who initiated chlorthalidone or hydrochlorothiazide during this period. Data were analyzed from December 2019 through September 2020.

**Exposures:**

New chlorthalidone users were matched 1:4 with new hydrochlorothiazide users by a high-dimensional propensity score. Time-to-event models accounting for competing risks examined the associations between chlorthalidone vs hydrochlorothiazide use and the outcomes of interest overall and within estimated glomerular filtration rate (eGFR) categories (≥60, 45-59, and <45 mL/min/1.73 m^2^).

**Main Outcomes and Measures:**

The outcomes of interest were adverse kidney events (ie, eGFR decline ≥30%, dialysis, or kidney transplantation), cardiovascular events (composite of myocardial infarction, coronary revascularization, heart failure, or atrial fibrillation), all-cause mortality, and electrolyte anomalies (ie, sodium or potassium levels outside reference ranges).

**Results:**

After propensity score matching, the study cohort included 12 722 adults (mean [SD] age, 74 [7] years; 7063 [56%] women; 5659 [44%] men; mean [SD] eGFR, 69 [19] mL/min/1.73 m^2^), including 2936 who received chlorthalidone and 9786 who received hydrochlorothiazide. Chlorthalidone use was associated with a higher risk of eGFR decline of 30% or greater (hazard ratio [HR], 1.24 [95% CI, 1.13-1.36]) and cardiovascular events (HR, 1.12 [95% CI, 1.04-1.22]) across all eGFR categories compared with hydrochlorothiazide use. Chlorthalidone use was also associated with a higher risk of hypokalemia compared with hydrochlorothiazide use, which was more pronounced among those with higher eGFR (eGFR ≥60 mL/min/1.73 m^2^: HR, 1.86 [95% CI, 1.67-2.08]; eGFR 45-59 mL/min/1.73 m^2^: HR, 1.57 [95% CI, 1.25-1.96]; eGFR <45 mL/min/1.73 m^2^: HR, 1.10 [95% CI, 0.84-1.45]; *P* for interaction = .001). No significant differences were observed between chlorthalidone and hydrochlorothiazide for dialysis or kidney transplantation (HR, 1.44 [95% CI, 0.88-2.36]), all-cause mortality (HR, 1.10 [95% CI, 0.93-1.29]), hyperkalemia (HR, 1.05 [95% CI, 0.79-1.39]), or hyponatremia (HR, 1.14 [95% CI, CI 0.98-1.32]).

**Conclusions and Relevance:**

This cohort study found that among older adults, chlorthalidone use was associated with a higher risk of eGFR decline, cardiovascular events, and hypokalemia compared with hydrochlorothiazide use. The excess risk of hypokalemia with chlorthalidone was attenuated in participants with reduced kidney function. Placed in context with prior observational studies comparing the safety and clinical outcomes associated with thiazide diuretics, these results suggest that there is no evidence to prefer chlorthalidone over hydrochlorothiazide.

## Key Points

**Question:**

What are the safety and clinical outcomes associated with chlorthalidone or hydrochlorothiazide use among older adults with varying levels of kidney function?

**Findings:**

In this cohort study of 12 722 older adults, chlorthalidone use was associated with a higher risk for eGFR decline of 30% or more, cardiovascular events, and hypokalemia compared with hydrochlorothiazide use. The excess risk of hypokalemia with chlorthalidone was attenuated in participants with reduced kidney function.

**Meaning:**

These findings suggest that there is no clear reason to prefer chlorthalidone over hydrochlorothiazide, although further randomized clinical trials may provide clarity into the comparative effectiveness of these 2 medications.

## Teaser

This cohort study compares the safety and clinical outcomes associated with chlorthalidone vs hydrochlorothiazide use among older adults with varying levels of kidney function.

## Introduction

Hypertension is the largest single contributor to morbidity and mortality worldwide.^1^ The prevalence of hypertension increases with age, and most hypertension-associated morbidity and mortality occur in older individuals.^2^ The health burden related to uncontrolled hypertension has diminished over time, owing to effective pharmacotherapy.^3^ As a class, thiazide diuretics effectively lower blood pressure (BP), reduce cardiovascular events, and are recommended as first-line antihypertensive agents.^4,5,6^ However, whether a specific thiazide is preferable in terms of safety and clinical outcomes remains unclear.

Hydrochlorothiazide is the most prescribed thiazide diuretic in North America,^7^ despite being shorter-acting^8^ and less potent (per milligram)^9,10,11^ than chlorthalidone. Limited head-to-head observational studies comparing these drugs have yielded mixed results. While older studies suggested that chlorthalidone was superior in controlling BP and reducing cardiovascular events,^12,13,14^ recent studies have demonstrated equivalency in cardiovascular risk reduction but a higher risk of adverse kidney outcomes and hypokalemia with chlorthalidone.^15,16^

Hypertension is nearly ubiquitous in individuals with chronic kidney disease (CKD), with a prevalence of more than 80%, including more than 50% requiring 3 or more antihypertensive medications.^17^ Despite early studies suggesting that thiazides have less diuretic and antihypertensive effects in CKD,^18,19^ recent studies have suggested that they remain effective in this population.^20,21,22,23^ Thiazides are now commonly prescribed to individuals with CKD.^24^ However, little is known about how chlorthalidone and hydrochlorothiazide compare among individuals with CKD. Herein, we conducted a large population-based retrospective cohort study of older adults to compare safety and clinical outcomes associated with chlorthalidone vs hydrochlorothiazide use across varying levels of kidney function.

## Methods

### Study Design and Setting

The use of data in this cohort study was authorized under section 45 of Ontario’s Personal Health Information Protection Act, which does not require review by a research ethics board or informed consent. We conducted a population-level, retrospective matched cohort study of older adults receiving medical treatment for hypertension from 2007 to 2015 in Ontario, Canada, using linked databases held at the ICES (eMethods in the Supplement). Ontario is Canada’s largest province, with more than 13 million residents, 16% of whom are aged 65 years or older.^25^ The reporting of this study follows Strengthening the Reporting of Observational Studies in Epidemiology (STROBE) reporting guideline and the Reporting of Studies Conducted Using Observational Routinely-Collected Health Data (RECORD) reporting guidelines for cohort studies.

### Cohort Definition

**Figure 1.**
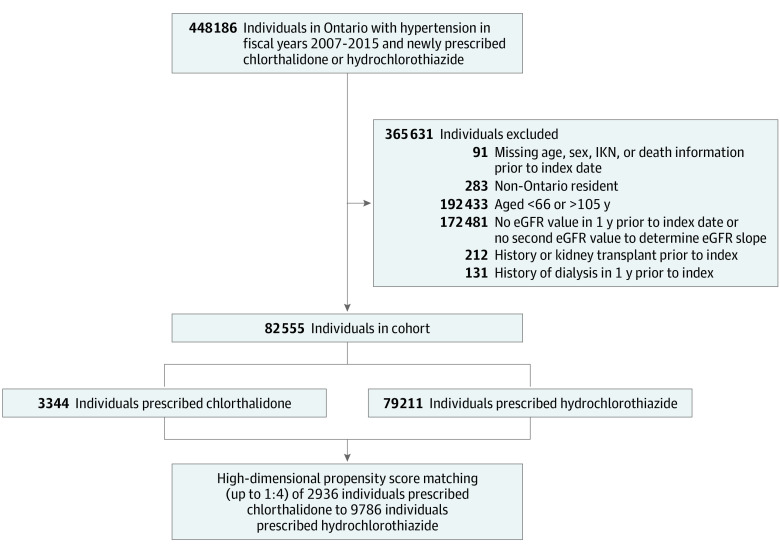
Flowchart for Cohort Assembly eGFR indicates estimated glomerular filtration rate; IKN, ICES Key Number.

All Ontario residents aged 66 years or older with a diagnosis of hypertension (defined by diagnostic code or dispensing of an antihypertensive medication), a first outpatient prescription (new user designation) dispensed for chlorthalidone or hydrochlorothiazide between April 2007 and March 2015, and a minimum of 2 estimated glomerular filtration rate (eGFR) measures were included (Figure 1; eTable 1 in the Supplement). We limited our cohort to adults aged 66 years or older because prescription drug information is only available for adults aged 65 years or older in Ontario. We initiated our cohort at age 66 years to allow for a 1-year look back period for pre-existing medications. Patients with a prior history of dialysis or kidney transplantation were excluded. eGFR was calculated using the CKD-EPI formula.^26^ Baseline eGFR was defined as the closest value within 1 year prior to index. A second eGFR, measured at least 60 days prior to the baseline eGFR and within 2 years of index, was required for study inclusion to determine eGFR slope prior to cohort entry. Patients were followed-up for up to 3 years after their index date (last follow-up date: March 31, 2016). The chlorthalidone or hydrochlorothiazide dispensing date served as the index date.

### Exposure

The study exposure was new use of chlorthalidone or hydrochlorothiazide within the accrual period. Each chlorthalidone user was matched with up to 4 hydrochlorothiazide users via a high-dimensional propensity score (HDPS).^27^ The HDPS is calculated by a computer algorithm designed for use in administrative databases that selects and ranks variables based on multiplicative bias testing (ie, an empirical method of variable selection). Given varying potencies of the study drugs, we further matched on thiazide dose. As chlorthalidone potency has been reported as 2- to 3-fold greater than hydrochlorothiazide,^9,10,11^ we dose-matched on a 1-mg:2-mg scheme based on categories of low-dose (chlorthalidone ≤12.5 mg/d matched to hydrochlorothiazide ≤25 mg/d), medium-dose (chlorthalidone 12.6-25 mg/d matched to hydrochlorothiazide 26-50 mg/d), and high-dose (chlorthalidone >25 mg/d matched to hydrochlorothiazide >50 mg/d).

### Outcomes

The outcomes of interest were adverse kidney events (ie, ≥30% eGFR decline, dialysis, or kidney transplantation), cardiovascular events (composite of acute myocardial infarction, coronary revascularization, heart failure, and atrial fibrillation), all-cause mortality, and electrolyte disturbances (ie, hypokalemia, hyperkalemia, and hyponatremia) (eTable 2 in the Supplement). For eGFR decline of 30% or more, a follow-up eGFR value was required; indexed participants without a follow-up eGFR value were excluded from this analysis. eGFR decline was defined using an eGFR from any time from more than 90 days to 3 years after the index date. Electrolyte disturbances were defined as hypokalemia (serum potassium ≤3.5 mEq/L [to convert to millimoles per liter, multiply by 1]), hyperkalemia (serum potassium ≥6.0 mEq/L), and hyponatremia (serum sodium ≤130 mEq/L [to convert to millimoles per liter, multiply by 1]). Recurrent outcomes were not considered. Death was a competing event for kidney, cardiovascular, and electrolyte outcomes. Crossover between chlorthalidone and hydrochlorothiazide use, emigration from Ontario, and conclusion of the study period were censoring events for all outcomes. Patients were followed-up until the earliest date among the specified outcome occurrence, emigration from Ontario, death, or the end of the study period (maximum 3 years).

### Statistical Analysis

We used standardized differences to assess covariate balance pre- and post-HDPS matching between chlorthalidone and hydrochlorothiazide users. This assesses differences between group means relative to the pooled SD, with a potentially important difference considered to be 0.1 or less.^28,29^ Participants dispensed chlorthalidone were matched (greedy, without replacement) up to 1:4 to participants dispensed hydrochlorothiazide on the logit of the HDPS (±0.2 of the SD) and according to study drug dose, sex, fiscal year of index (±2 years), eGFR (±10 mL/min/1.73 m^2^), heart failure, diabetes, loop diuretic use, and glucose-lowering agent use. Heart failure, diabetes, loop diuretic use, and glucose-lowering agent use were included owing to a relative imbalance after the initial HDPS match. Variables selected by the HDPS algorithm were visually inspected for clinical appropriateness and truncated to the top 201 covariates based on multiplicative bias ranking (eTable 3 in the Supplement). We calculated incidence rates for the outcomes of interest. We examined the associations between chlorthalidone or hydrochlorothiazide exposure with kidney, cardiovascular, and electrolyte events using Fine and Gray models to calculate subdistribution hazard ratios (HRs) with 95% CI based on an intention-to-treat design. These models accounted for the competing risk of death. To analyze all-cause mortality, we used Cox proportional hazards models. Within these models, we assessed for differential relative risk between eGFR categories (≥60, 45-59, and <45 mL/min/1.73 m^2^) and chlorthalidone or hydrochlorothiazide use for the outcomes of interest using an interaction term. Models were adjusted for baseline eGFR slope (prespecified) as well as use of angiotensin-converting enzyme (ACE) inhibitors, angiotensin receptor blockers (ARB), and calcium channel blockers (CCB) and nephrological care (added to the models to correct for imbalance of these variables between chlorthalidone and hydrochlorothiazide users that persisted following the final HDPS match). We conducted all analyses with SAS statistical software version 7.15 (SAS Institute). 95% CIs that did not overlap with 1.0 and 2-sided *P* < .05 were treated as statistically significant.

Additional analyses were conducted using a chlorthalidone to hydrochlorothiazide dose-matching scheme of 1 mg:3 mg based on categories of low-dose (chlorthalidone ≤12.5 mg/d matched to hydrochlorothiazide ≤37.5 mg/d), medium-dose (chlorthalidone 12.6-25 mg/d matched to hydrochlorothiazide 37.6-75 mg/d), and high-dose (chlorthalidone >25 mg/d matched to hydrochlorothiazide >75 mg/d). A second analysis was conducted censoring participants at drug discontinuation (ie, an as-treated design). A third analyses used propensity matching on number of antihypertensive agents (range, 1-3 agents), which consisted of the thiazide plus an ACE inhibitor, ARB, or CCB. A fourth analysis was conducted by restricting to thiazide monotherapy. Data were analyzed from December 2019 to September 2020.

## Results

### Baseline Characteristics

After HDPS matching, the analysis cohort consisted of 12 722 older adults (mean [SD] age, 74 [7] years; 7063 [56%] women; 5659 [44%] men; mean [SD] eGFR, 69 [19] mL/min/1.73 m^2^), including 2936 newly dispensed a prescription for chlorthalidone and 9786 newly dispensed hydrochlorothiazide (Figure 1) (Table). Participants using chlorthalidone had higher rates of ACE inhibitor use, CCB use, and nephrological care, while participants using hydrochlorothiazide had higher rates of ARB use. Mean follow-up times for all study outcomes are displayed in eTable 4 in the Supplement. Chlorthalidone and hydrochlorothiazide were more commonly prescribed as add-on therapy (chlorthalidone: 2647 participants [90%]; hydrochlorothiazide: 9050 participants [93%]) rather than as monotherapy (chlorthalidone: 289 participants [10%]; hydrochlorothiazide: 736 participants [8%]).

**Table.** Baseline Study Characteristics of Propensity Score–Matched Patients Receiving Chlorthalidone or Hydrochlorothiazide

Characteristic	No. (%)		
	Chlorthalidone (n = 2936)	Hydrochlorothiazide (n = 9786)	Standardized differences^a^
Age, mean (SD), y	74 (7)	74 (7)	0.013
Sex			
Women	1599 (54)	5464 (56)	0.000
Men	1337 (46)	4322 (44)	0.000
Income quintile			
1 (lowest)	590 (20)	1960 (20)	0.014
2	635 (22)	2135 (22)	0.016
3	586 (20)	2008 (21)	0.006
4	566 (19)	1893 (19)	0.005
5 (highest)	559 (19)	1790 (18)	0.027
Rural residence^b^	329 (11)	1032 (11)	0.032
Year of index date			
2007	0	≤5 (<1)	0.023
2008	32 (1)	115-120 (1)	0.018
2009	162 (6)	592 (6)	0.023
2010	328 (11)	1347 (14)	0.077
2011	442 (15)	1581 (16)	0.030
2012	679 (23)	1814 (19)	0.107
2013	644 (22)	1905 (19)	0.064
2014	649 (22)	2427 (25)	0.060
Total antihypertensive medications, No.			
1	289 (10)	736 (7)	0.117
2	603 (21)	2186 (22)	0.014
3	789 (27)	2793 (29)	0.010
4	742 (25)	2370 (24)	0.001
5	378 (13)	1251 (13)	0.059
6	117 (4)	407 (4)	0.061
7	18 (1)	43 (1)	0.006
eGFR, mL/min/1.73 m^2^^c^			
Mean (SD)	68.8 (18.9)	69.1 (18.4)	0.020
Category			
≥60	2022 (69)	7295 (74)	0.017
45-59	515 (18)	1628 (17)	0.007
<45	399 (14)	863 (9)	0.032
Comorbidities^d^			
Coronary artery disease	792 (27)	2388 (24)	0.005
Myocardial infarction	114 (4)	256 (3)	0.031
CABG	58 (2)	153 (2)	0.009
Heart failure	290 (10)	606 (6)	0.000
Atrial fibrillation	168 (6)	475 (5)	0.009
Arrhythmia	255 (9)	695 (7)	0.007
Ischemic stroke	92 (3)	182 (2)	0.072
Peripheral vascular disease	56 (2)	121 (1)	0.034
Diabetes	1322 (45)	4122 (42)	0.000
COPD	99 (3)	299 (3)	0.021
Chronic liver disease	136 (5)	390 (4)	0.028
Major cancer	385 (13)	1281 (13)	0.003
Seizure	25 (1)	54 (1)	0.020
Osteoporosis	18 (1)	59 (1)	0.005
Medications^e^			
ACE inhibitors	1239 (42)	3066 (31)	0.191
ARBs	847 (29)	3845 (39)	0.228
Calcium channel blockers	1288 (44)	3526 (36)	0.106
β-Blockers	1104 (38)	3060 (31)	0.071
Loop diuretic	233 (8)	406 (4)	0.000
α-Blocker	117 (4)	252 (3)	0.042
Nitrates	156 (5)	475 (5)	0.024
Clonidine	18 (1)	24 (1)	0.051
Antiarrhythmics	39 (1)	115 (1)	0.003
Clopidogrel	177 (6)	446 (5)	0.037
Statins	1724 (59)	5575 (57)	0.006
Glucose-lowering medications	935 (32)	2822 (29)	0.000
Antipsychotics	78 (3)	236 (2)	0.002
Health services^f^			
Family physician	2869 (98)	9591 (98)	0.026
Nephrologist	588 (20)	835 (9)	0.251
Cardiologist	1582 (54)	4595 (47)	0.088

Abbreviations: ACE, angiotensin converting enzyme; ARB, angiotensin II receptor blocker; CABG, coronary artery bypass graft; COPD, chronic obstructive pulmonary disease; eGFR, estimated glomerular filtration rate.

^a^ Weighted standardized differences were used to account for the variable number of participants receiving hydrochlorothiazide matched to each participant receiving chlorthalidone.^29^ The crude statistics given in the post–propensity score-matching cohort for each group do not correspond to the weighted standardized differences. Standardized differences are less sensitive to sample size than traditional hypothesis tests. They provide a measure of the difference between groups divided by the pooled SD; a value greater than 10% is interpreted as a meaningful difference between groups.^28^

^b^ Rural was defined as residing in a location with population of fewer than 10 000 people.

^c^ Kidney function was defined at baseline as the eGFR value closest to the index date within 1 year up to and including the index date.

^d^ Comorbidities were ascertained in the 5 years prior to cohort entry.

^e^ Medication use was ascertained in the 120 days prior to cohort entry.

^f^ Health service utilization was ascertained in the 1 year prior to cohort entry.

### Adverse Kidney Events

**Figure 2.**
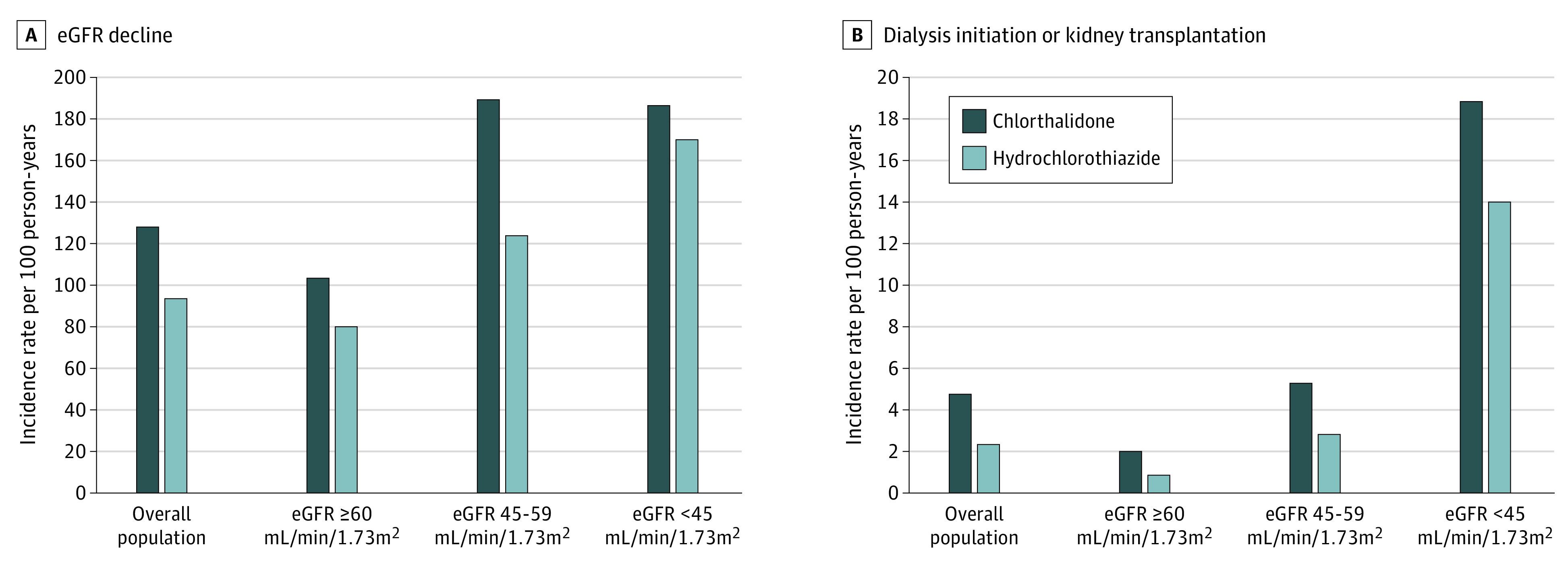
Adverse Kidney Events eGFR indicates estimated glomerular filtration rate.

Chlorthalidone use was associated with a higher risk of eGFR decline of 30% or greater compared with hydrochlorothiazide use (128 [95% CI, 118-138] events per 1000 person-years vs 93.7 [95% CI, 89.3-98.1] events per 1000 person-years; HR, 1.24 [95% CI, 1.13-1.36]) (Figure 2A). There was no modification associated with eGFR category in the association of chlorthalidone or hydrochlorothiazide use with eGFR decline of 30% or more (eTable 5 in the Supplement). For dialysis or kidney transplantation, there was no significant difference in risk between chlorthalidone and hydrochlorothiazide use (4.75 [95% CI, 3.08-6.42] events per 1000 person-years vs 2.29 [95% CI, 1.69-2.90] events per 1000 person-years; HR, 1.44 [95% CI, 0.88-2.36]) (Figure 2B) with no modification of association by eGFR category (eTable 5 in the Supplement).

### Cardiovascular Events

**Figure 3.**
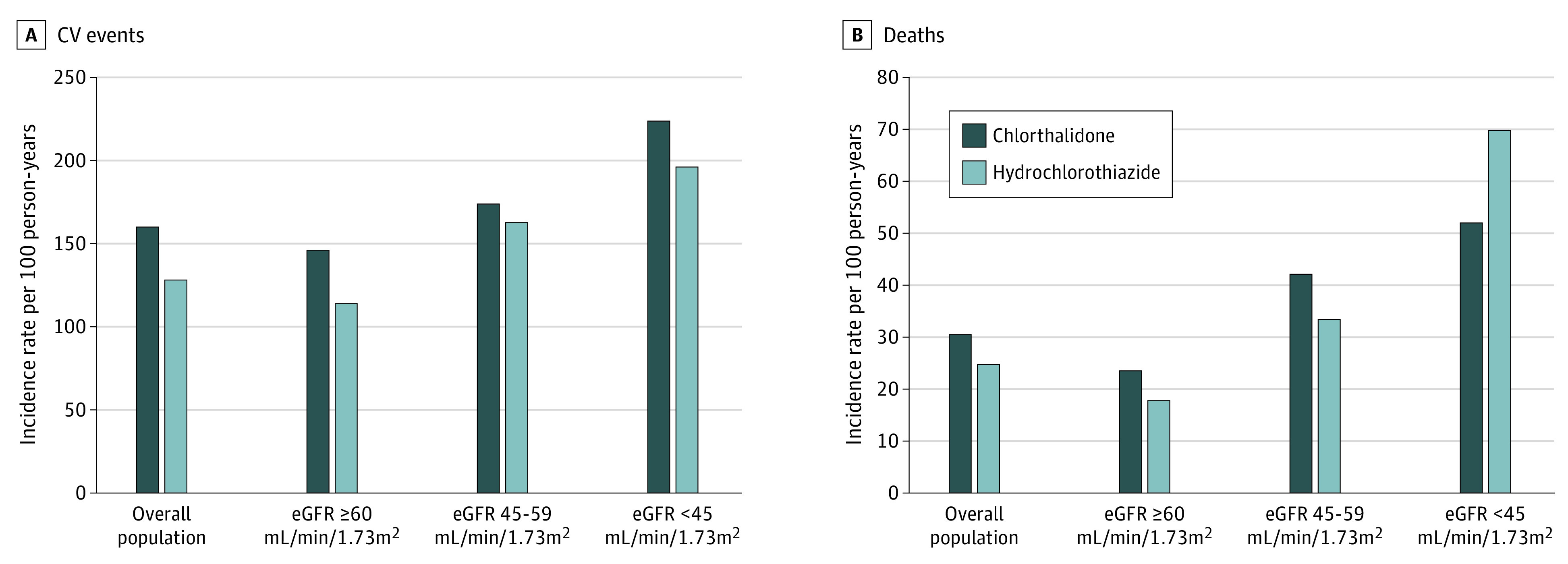
Cardiovascular (CV) Events and All-Cause Mortality eGFR indicates estimated glomerular filtration rate.

Chlorthalidone use was associated with a higher risk of cardiovascular events compared with hydrochlorothiazide use (160 [95% CI, 150-171] events per 1000 person-years vs 128 [95% CI, 123-133] events per 1000 person-years; HR, 1.12 [95% CI, 1.04-1.22]) (Figure 3A). There was no modification of association by eGFR category (eTable 5 in the Supplement).

### All-Cause Mortality

There was no significant difference in all-cause mortality between chlorthalidone and hydrochlorothiazide groups (30.5 [95% CI, 26.3-34.8] events per 1000 person-years vs 24.7 [95% CI, 22.8-26.7] events per 1000 person-years; HR, 1.10 [95% CI, 0.93-1.29]) (Figure 3B). However, among participants with eGFR of 60 mL/min/1.73 m^2^ or greater, chlorthalidone was associated with a higher all-cause mortality risk compared with hydrochlorothiazide (23.5 [95% CI, 19.1-28.0] events per 1000 person-years vs 17.8 [95% CI, 15.9-19.7] events per 1000 person-years; HR, 1.27 [95% CI, 1.02-1.58]). In contrast, among participants with eGFR of 60 mL/min/1.73 m^2^ or less, there was no significant difference in all-cause mortality risk. eGFR category was associated with modifying the association between chlorthalidone or hydrochlorothiazide use and all-cause mortality (eTable 5 in the Supplement).

### Electrolyte Disturbances

**Figure 4.**
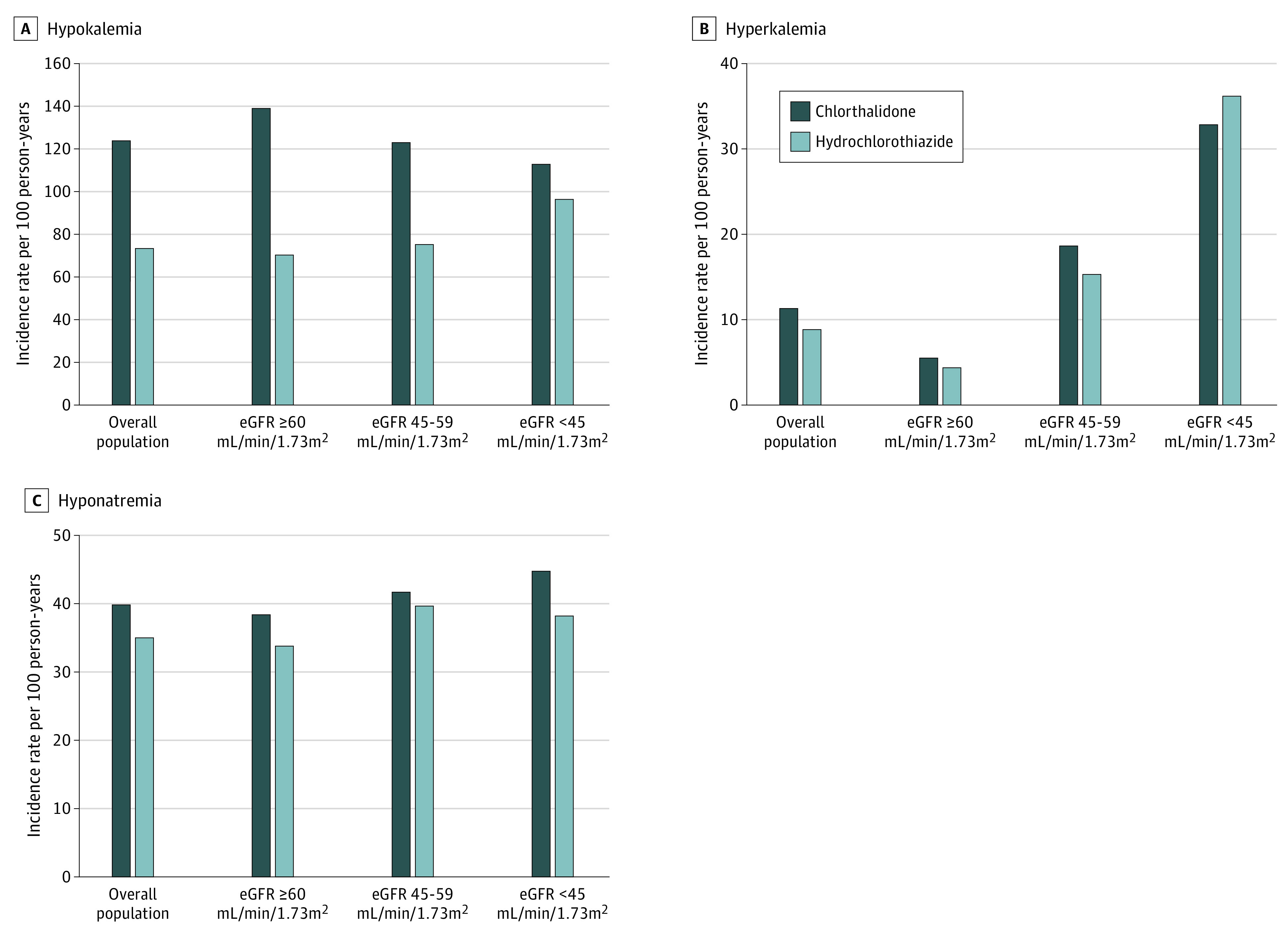
Electrolyte Disturbances eGFR indicates estimated glomerular filtration rate.

Chlorthalidone use was associated with a higher risk of hypokalemia compared with hydrochlorothiazide use (133 [95% CI, 123-142] events per 1000 person-years vs 73 [95% CI, 70-77] events per 1000 person-years; HR, 1.70 [95% CI, 1.55-1.87]) (Figure 4A). The increased risk of hypokalemia associated with chlorthalidone was more prominent in patients with higher baseline kidney function (eGFR ≥60 mL/min/1.73 m^2^: 139 [5%CI 127-151] events per 1000 person-years vs 70.2 [95% CI, 66.1-74.4] events per 1000 person-years; HR, 1.86 [95% CI, 1.67-2.08]; eGFR 45-59 mL/min/1.73 m^2^: 123 [95% CI, 101-145] events per 1000 person-years vs 75.4 [95% CI, 66.2-84.6] events per 1000 person-years; HR, 1.57 [95% CI, 1.25-1.96]; eGFR <45 mL/min/1.73 m^2^: 113 [95% CI, 88-137] events per 1000 person-years vs 96.8 [95% CI, 82.4-111.3] events per 1000 person-years; HR, 1.10 [95% CI, 0.84-1.45]; *P* for interaction = .001). There was no significant difference between chlorthalidone and hydrochlorothiazide groups in risk of hyperkalemia (11.4 [95% CI, 8.7-14.0] events per 1000 person-years vs 8.84 [95% CI, 7.62-10.06] events per 1000 person-years; HR, 1.05 [95% CI, 0.79-1.39]) (Figure 4B) or hyponatremia (39.8 [95% CI, 34.7-44.9] events per 1000 person-years vs 35.1 [95% CI, 32.7-37.6] events per 1000 person-years; HR, 1.14 [95% CI, 0.98-1.32]) (Figure 4C), with no association modification by eGFR category (eTable 5 in the Supplement).

### Additional Analyses

Models incorporating a chlorthalidone to hydrochlorothiazide dose-matching scheme of 1 mg to 3 mg, censoring at drug discontinuation (as-treated), matching on antihypertensive medication use, and restricting to thiazide monotherapy showed similar estimated associations (eTable 5 in the Supplement). For the as-treated analysis, the mean (SD) time using the study drug was 318 (334) days for chlorthalidone and 375 (360) days for hydrochlorothiazide.

## Discussion

In this population-based cohort study of individuals aged 66 years and older, we found that chlorthalidone use was associated with a higher risk of eGFR decline, cardiovascular events, and hypokalemia compared with hydrochlorothiazide use. The increased risk for hypokalemia associated with chlorthalidone vs hydrochlorothiazide was attenuated in patients with reduced kidney function.

Our results expand on prior studies comparing safety and clinical outcomes associated with chlorthalidone and hydrochlorothiazide use. First, to our knowledge, no prior studies have compared chlorthalidone and hydrochlorothiazide head-to-head across levels of kidney function. As thiazides are increasingly prescribed in CKD,^24^ understanding their differential outcomes associated with level of kidney function may allow for more personalized hypertension care. Second, most prior studies comparing chlorthalidone and hydrochlorothiazide have not accounted for their differing potencies.^12,15,30^ Chlorthalidone is 2- to 3-fold more potent (per milligram) than hydrochlorothiazide.^9,10,11^ We comprehensively examined outcomes between adults using chlorthalidone or hydrochlorothiazide on both 1 mg:2 mg and 1 mg:3 mg dose-matching schemes with similar findings. Third, several prior studies limited the comparison between chlorthalidone and hydrochlorothiazide to their use as first-line agents,^15,16^ whereas they are recommended and commonly used as add-on therapy.^4,5,6^ Non–first-line thiazide use is particularly relevant in CKD, in which alternative agents, such as ACE inhibitors and ARBs, have well-established protective associations for the kidneys and are preferentially prescribed as first-line therapy. By allowing for thiazides as first-line or add-on therapy, our study design is more reflective of real-world practice.^31^

Our finding of a higher risk of kidney disease progression associated with chlorthalidone vs hydrochlorothiazide correlates with the results from a recent large observational cohort study by Hripcsak et al,^16^ which reported higher rates of acute kidney injury and CKD with chlorthalidone vs hydrochlorothiazide monotherapy. Our study demonstrates that the higher rates of adverse kidney events associated with chlorthalidone vs hydrochlorothiazide persist even after dose matching. Although testing for association modification of baseline eGFR on the association between chlorthalidone or hydrochlorothiazide use and eGFR decline of 30% or more did not meet the level of significance, it is noteworthy that among patients with a baseline eGFR less than 45 mL/min/1.73 m^2^, there was no difference in risk for eGFR decline of 30% or greater. This may relate to reduced drug activity at the level of the nephron in more advanced CKD.^18^ Therefore, the risk of adverse kidney events associated with chlorthalidone vs hydrochlorothiazide may be more pronounced in patients with more preserved kidney function.

In regard to cardiovascular outcomes, to our knowledge, there are no randomized clinical trials directly comparing chlorthalidone and hydrochlorothiazide. The best available evidence comes via observational studies with mixed results. Dorsch et al^12^ performed a retrospective analysis of the Multiple Risk Factor Intervention Trial and found lower cardiovascular event rates among participants receiving chlorthalidone vs hydrochlorothiazide. Similarly, a network meta-analysis comparing the 2 agents showed that chlorthalidone use was associated with lower cardiovascular event risk.^30^ Conversely, several recent population-based cohort studies have contrasted these findings. Dhalla et al^15^ and Hripcsak et al^16^ compared chlorthalidone vs hydrochlorothiazide as first-line antihypertensive agents and found no significant difference in cardiovascular outcomes.

In contrast, our study found that chlorthalidone use was associated with a higher risk for cardiovascular events compared with hydrochlorothiazide use. However, we cannot draw a conclusion about causality, particularly given the mixed results from prior studies, combined with the inherent limitations of overinterpreting administrative data. Notably, in our additional analyses matching based on antihypertensive medication use and restricting to thiazide monotherapy, we found no association between chlorthalidone vs hydrochlorothiazide use and cardiovascular events. At a minimum, our results suggest that among older adults, chlorthalidone use was not associated with a reduced risk for cardiovascular events compared with hydrochlorothiazide use. An ongoing randomized clinical trial through the Veterans Health Administration comparing cardiovascular events between chlorthalidone and hydrochlorothiazide will hopefully provide further clarity.^32^

We did observe a higher risk of hypokalemia associated with chlorthalidone use compared with hydrochlorothiazide use, which is consistent with prior observational studies.^15,16^ In our primary analysis, we found that chlorthalidone vs hydrochlorothiazide use was associated with a HR for hypokalemia of 1.70 (95% CI, 1.55-1.1.87), which is actually lower than that reported in other observational studies.^15,16^ This may be associated with the intention-to-treat design, as our as-treated sensitivity analysis found a HR for hypokalemia more on par those prior studies. Our study expands on these prior works by demonstrating that this increased risk for hypokalemia persists even after dose matching between chlorthalidone and hydrochlorothiazide. In addition, we now demonstrate that the excess risk for hypokalemia associated with chlorthalidone was attenuated in participants with reduced kidney function. Perhaps this reflects reduced drug concentrations at the nephron level in participants with CKD or reduced baseline potassium excretion as kidney function declines.

What are some potential clinical implications of a higher risk of hypokalemia associated with chlorthalidone vs hydrochlorothiazide? Numerous studies have demonstrated that hypokalemia in patients with hypertension receiving diuretics is associated with an increased risk of cardiovascular events and death.^33,34,35,36^ In our study, chlorthalidone use was associated with a 70% increased risk of hypokalemia compared with hydrochlorothiazide, which was observed primarily by participants with preserved eGFR. One could postulate that the higher rates of hypokalemia associated with chlorthalidone from our study (particularly among those with preserved eGFR) may have contributed to our findings regarding cardiovascular events and mortality. Notably, among participants with eGFR less than 45 mL/min/1.73 m^2^, in whom there was no significant difference in hypokalemia, we also found no significant difference in cardiovascular events or mortality. However, prospective or interventional studies will be necessary to more fully understand this link.

### Limitations

This study has some limitations. Our results must be interpreted within the context of the study design. First, this study is observational involving administrative health care data; therefore, we were able to identify association but not causation. The use of HDPS for matching the chlorthalidone and hydrochlorothiazide groups theoretically should reduce observed confounding and examines proxies associated with disease severity. HDPS has been shown to improve covariate balance and minimize confounding from observed covariates compared with other forms of matching.^27^ After HDPS-matching, several imbalances remained (ACEI, ARB, and CCB use and nephrological care). We adjusted for these variables within our analyses and performed sensitivity analyses with consistent results; however, we acknowledge that residual confounding may still remain. We also followed recommended principles for research using administrative data, including prespecifying the cohort creation and analysis plan, studying multiple outcomes simultaneously, reporting on all prespecified outcomes, and incorporating a network of databases.^37^ Second, BP measurement data was not available in our datasets. However, the total numbers of antihypertensive medications prescribed, for which we had accurate and reliable data, were comparable between groups. Despite this, given the lack of BP data and differential prescription patterns of chlorthalidone vs hydrochlorothiazide, we cannot rule out potential residual confounding by indication. Also, given the study design we were able to account for antihypertensive prescription dispensing but not necessarily adherence which may impact clinical outcomes.^38^ Third, the study index period was from 2007 to 2015, which could present an element of historical bias; however, antihypertensive treatment regimens did not change significantly over this period.^39^ Fourth, our inclusion and exclusion criteria (eg, requiring 2 eGFR values prior to index) reduced the population size we were able to study, which may limit the generalizability of our findings.

## Conclusions

In this population-based cohort study of older adults, we found that chlorthalidone use was associated with a higher risk of eGFR decline, cardiovascular events, and hypokalemia compared with hydrochlorothiazide use. The excess risk of hypokalemia associated with chlorthalidone was attenuated in participants with reduced kidney function. Placed in context with prior observational studies comparing the safety and clinical outcomes associated with thiazide diuretics, these results suggest that there is no clear reason to prefer chlorthalidone over hydrochlorothiazide.

## Notes

Supplement. **eMethods.** Full Details on Data Sources **eTable 1.** Full Unmatched Cohort **eTable 2.** Databases and Coding Definitions for Inclusion and Exclusion Criteria, Outcomes, and Baseline Characteristics **eTable 3.** List of Covariates Included in High Dimensional Propensity Score Matching Algorithm in Rank Order **eTable 4.** Mean Follow-up Time Among Chlorthalidone and Hydrochlorothiazide Users Overall and by eGFR Category **eTable 5.** Sensitivity Analyses of Associations of Chlorthalidone vs Hydrochlorothiazide With Study Outcomes
eReferences
