# Errors in Figures 2, 3, and 4

**DOI:** 10.1001/jamanetworkopen.2023.17603

**Published:** 2023-05-22

In the Original Investigation titled “Comparison of Clinical Outcomes and Safety Associated With Chlorthalidone vs Hydrochlorothiazide in Older Adults With Varying Levels of Kidney Function,”^1^ published September 15, 2021, there were errors in the y-axis labels in Figures 2, 3, and 4. These should have read “Incidence rate per 1000 person-years.” This article has been corrected.^1^
