# Supplementary material for: Comparison of Clinical Outcomes and Safety Associated With Chlorthalidone vs Hydrochlorothiazide in Older Adults With Varying Levels of Kidney Function
Source: JAMA Netw Open. 2021 Sep 15;4(9):e2123365. doi: 10.1001/jamanetworkopen.2021.23365 (PMC8444030; doi:10.1001/jamanetworkopen.2021.23365)
Supplement: Supplement. — eMethods. Full Details on Data Sources eTable 1. Full Unmatched Cohort eTable 2. Databases and Coding Definitions for Inclusion and Exclusion Criteria, Outcomes, and Baseline Characteristics eTable 3. List of Covariates Included in High Dimensional Propensity Score Matching Algorithm in Rank Order eTable 4. Mean Follow-up Time Among Chlorthalidone and Hydrochlorothiazide Users Overall and by eGFR Category eTable 5. Sensitivity Analyses of Associations of Chlorthalidone vs Hydrochlorothiazide With Study Outcomes eReferences [file jamanetwopen-e2123365-s001.pdf]

Supplemental Online Content

Edwards C, Hundemer GL, Petrcich W, et al. Comparison of clinical outcomes and safety associated with chlorthalidone vs hydrochlorothiazide in older adults with varying levels of kidney function. JAMA Netw Open. 2021;4(9):e2123365. doi:10.1001/jamanetworkopen.2021.23365

eMethods. Full Details on Data Sources

eTable 1. Full Unmatched Cohort

eTable 2. Databases and Coding Definitions for Inclusion and Exclusion Criteria, Outcomes, and Baseline Characteristics

eTable 3. List of Covariates Included in High Dimensional Propensity Score Matching Algorithm in Rank Order

eTable 4. Mean Follow-up Time Among Chlorthalidone and Hydrochlorothiazide Users Overall and by eGFR Category

eTable 5. Sensitivity Analyses of Associations of Chlorthalidone vs Hydrochlorothiazide With Study Outcomes

eReferences

This supplemental material has been provided by the authors to give readers additional information about their work.

© 2021 Edwards C et al. *JAMA Network Open.*

eMethods. Full Details on Data Sources

We ascertained patient characteristics, medication data, and outcome data from de-identified, linked databases housed at ICES. Demographics and vital status information were obtained from the Ontario Registered Persons Database. Medication information was obtained from the Ontario Drug Benefit Claims (ODB) database. This database contains highly accurate records of all outpatient prescriptions dispensed to patients ≥65 years of age, with an error rate of <1%.[1] Diagnostic and procedural information from all hospitalizations were determined using the Canadian Institute for Health Information Discharge Abstract Database (CIHI-DAD). Diagnostic information from emergency room and day surgery visits was determined using the National Ambulatory Care Reporting System (NACRS). Information was also obtained from the Ontario Health Insurance Plan (OHIP) database, which contains all health claims for inpatient and outpatient physician services. Whenever possible, we defined patient characteristics and outcomes using validated codes (**eTable 2**). Laboratory information is contained in the Ontario Laboratory Information System (OLIS) that captures laboratory tests for patients in Ontario. The databases were complete for all variables used except for rural location and income, which were missing in <0.5% of patients. The only reason for lost follow-up was emigration from the province which occurs in <0.5% of residents annually.[2]

© 2021 Edwards C et al. *JAMA Network Open.*

eTable 1. Full Unmatched Cohort

Characteristic	No. (%)		Standardized differencesa
	Chlorthalidone (n = 3344)	Hydrochlorothiazide (n = 79 211)	
Age, mean (SD), y	74 (7)	75 (7)	0.063
Sex			
Women	1776 (53)	46 056 (58)	0.101
Men	1568 (47)	33 155 (42)	0.101
Income quintile			
1 (lowest)	684 (20)	15 708 (20)	0.015
2	720 (22)	16 561 (21)	0.015
3	679 (20)	15 906 (20)	0.005
4	636 (19)	15 664 (20)	0.019
5 (highest)	618 (19)	15 173 (19)	0.017
Rural residencec	367 (11)	9444 (12)	0.030
Year of index date			
2007	0 (0)	87 (0)	0.047
2008	39 (1)	2782 (4)	0.156
2009	189 (6)	9137 (12)	0.211
2010	376 (11)	13 754 (17)	0.175
2011	490 (15)	13 331 (17)	0.060
2012	761 (23)	12 738 (16)	0.169
2013	727 (22)	12 947 (16)	0.138
2014	762 (23)	14 435 (18)	0.113
Total antihypertensive medications, No.			
1	296 (9)	9501 (12)	0.103
2	635 (19)	20 662 (26)	0.170
3	862 (26)	22 291 (28)	0.053
4	881 (26)	16 475 (21)	0.131
5	468 (14)	7902 (10)	0.124
6	173 (5)	2120 (3)	0.129
7	29 (1)	260 (0)	0.07
eGFR, mL/min/1.73 m2			
Mean (SD) d	66.2 (20.7)	71.1 (17.6)	0.257
Categoryd			
≥60	2137 (64)	58 874 (74)	0.227
45-59	590 (18)	13 011 (16)	0.032
<45	617 (18)	7326 (9)	0.269
Comorbiditiese			
Coronary artery disease	969 (29)	20 444 (26)	0.071
Myocardial infarction	159 (5)	2672 (3)	0.070
CABG	84 (3)	1517 (2)	0.041
Heart failure	466 (14)	7923 (10)	0.121
Atrial fibrillation	235 (7)	5073 (6)	0.025
Arrhythmia	347 (10)	7445 (9)	0.033
Ischemic stroke	109 (3)	2110 (3)	0.035
Peripheral vascular disease	73 (2)	1230 (2)	0.047

© 2021 Edwards C et al. *JAMA Network Open.*

Diabetes	1585 (47)	32 250 (41)	0.135
COPD	139 (4)	3306 (4)	0.001
Chronic liver disease	167 (5)	3172 (4)	0.048
Major cancer	456 (14)	11 304 (14)	0.018
Seizure	31 (1)	602 (1)	0.018
Osteoporosis	24 (1)	818 (1)	0.034
Medicationsf			
ACE inhibitors	1444 (43)	29 212 (37)	0.129
ARBs	984 (29)	23 483 (30)	0.005
Calcium channel blockers	1546 (46)	25 125 (32)	0.301
β blockers	1332 (40)	23 480 (30)	0.215
Loop diuretic	434 (13)	5231 (7)	0.216
α blocker	168 (5)	1843 (2)	0.144
Nitrates	204 (6)	4363 (6)	0.025
Clonidine	25 (1)	219 (0)	0.066
Antiarrhythmics	53 (2)	1119 (1)	0.014
Clopidogrel	227 (7)	4361 (6)	0.053
Statins	2002 (60)	43 554 (55)	0.099
Glucose-lowering medications	1122 (34)	20 763 (26)	0.161
Antipsychotics	93 (3)	2506 (3)	0.023
Health servicesg			
Family physician	3261 (98)	77 720 (98)	0.041
Nephrologist	799 (24)	6968 (9)	0.417
Cardiologist	1865 (56)	38 375 (48)	0.147

© 2021 Edwards C et al. *JAMA Network Open.*

eTable 2. Databases and Coding Definitions for Inclusion and Exclusion Criteria, Outcomes, and Baseline Characteristics

Characteristic/Condition	Database	Codes
**Inclusion Criteria**		
Hypertension (from fiscal 1997 onward):<br>Defined as<br>1. 1 hospital admission with a hypertension diagnosis<br>2. OHIP claim with hypertension diagnosis, followed within 2 years by either an OHIP claim or hospital admission with a hypertension diagnosis<br>3. Rx for BP lowering medication in accrual period	CIHI-DAD<br><br><br>OHIP	**ICD9: ICD9:** 401, 402, 403, 404, 405<br><br>**ICD10:** I10, I11, I12, I13, I15<br><br>**OHIP DX:** 401, 402, 403
BP- Lowering Medication<br>S_BBL<br>S_ACE<br>S_ARB<br>S_THD<br>S_CCB	ODB	
**Exclusion Criteria**		
Age (<66 or >105)	RPDB	N/A
Hydrochlorothiazide or Chlorthalidone in the 180 days prior to index date	ODB	
Dialysis in the year up to index date	DAD-SDS<br>or<br>NACRS<br><br>OHIP	**CCI:** 1PZ21<br>**ICD10:** Z49, Z992<br><br><br>**OHIP FEE:**<br><br>R849 G323 G325 G326 G860 G862 G865 G863 G866<br><br>G330 G331 G333 G861 G082 G083 G085 G090 G091 G092 G093 G094 G095 G096 G294 G295 G864 H540 H740
Kidney Transplantation ever on or before index date	DAD-SDS<br>or<br>NACRS<br><br>OHIP	**CCI:** 1PC85<br>**CCP:** 675<br><br><br>**OHIP FEE:**<br>E762 E769 E771 G408 G409 G412 S434 S435 S437 Z631
**Exposure**		
Hydrochlorothiazide<br>or<br>Chlorthalidone	ODB	

© 2021 Edwards C et al. *JAMA Network Open.*

**Outcomes**		
>30% Decline in eGFR from baseline value<br>If decline greater than 30 % occurs, the earliest date after index date on which it occurs is the outcome date.	OLIS<br><br>DAD-SDS and NACRS to remove inpatient lab values	**OLIS:** Observation code 14682-9 (date part of OBSERVATIONDATETIME over 90 days after index date). CKD-EPI equation used to calculate eGFR from cleaned SCr values<br><br>Lab values were kept for assessment of this outcome so long as they did not fall within a DAD/SDS hospitalization or a NACRS ED visit
Dialysis or Kidney Transplantation	DAD-SDS or NACRS<br><br>OHIP<br><br>DAD-SDS or NACRS<br><br>OHIP	**Kidney transplant:**<br>**CCI:** 1PC85<br><br>**OHIP FEE:** E762 E769 E771 G408 G409 G412 S434 S435 S437 Z631<br>**Dialysis:**<br>**ICD10:** Z49 Z992<br>**CCI:** 1PZ21<br><br>**OHIP FEE:** R849 G323 G325 G326 G860 G862 G865 G863 G866 G330 G331 G333 G861 G082 G083 G085 G090 G091 G092 G093 G094 G095 G096 G294 G295 G864 H540 H740
Cardiac Events:<br>Atrial Fibrillation<br>Congestive Heart Failure<br>Coronary Artery Disease<br>Myocardial Infarction<br>CABG<br>PCI	DAD-SDS or NACRS<br><br>OHIP	**ICD10:** I48 I099 I420 I425 I426 I427 I428 I429 I43 I500 I501 I509 I255 J81 I21 I22 Z955 T822 I21 I22<br>**CCI:** 1HP53 1HP55 1HZ53GRFR 1HZ53LAFR 1HZ53SYFR 1IJ50 1IJ76 1IJ50 1IJ57GQ 1IJ54GQAZ<br><br>**OHIP FEE:** R701 R702 Z429 R741 R742 R743 G298 E646 E651 E652 E654 E655 Z434 Z448 E645 G262<br>**OHIP DX:** 428 410 412
Hypokalemia (earliest hypokalemia episode defined as occurring on date of an OLIS lab value showing hypokalemia)	OLIS	**OLIS:** OLIS observation code 2823-3 serum potassium ≤ 3.5.
Hyperkalemia (earliest hyperkalemia episode defined as occurring on date of an OLIS lab value showing hyperkalemia)	OLIS	**OLIS:** OLIS observation code 2823-3 serum potassium ≥ 6.0.
Hyponatremia (earliest hyponatremia episode defined as occurring on date of an OLIS lab value showing hyponatremia)	OLIS	**OLIS:** OLIS observation code 2951-2 [Ser/Pla] sodium ≤ 130.
Mortality	RPDB	
**Baseline Characteristics**		
Age, Sex, Income Quintile, Rurality	RPDB	

© 2021 Edwards C et al. *JAMA Network Open.*

Index fiscal year	ODB	Fiscal year of dispensing date of index prescription (ODB SERVDATE)
Number of different hypertension medications prescribed from hypertension diagnosis date (inclusion step) up to index date	ODB	
Kidney Function (SCr, eGFR)	OLIS	**Baseline eGFR:**<br><br>Observation code 14682-9 (Serum creatinine)<br>Converted to eGFR using CKD-EPI equation<br>Deleted if date part of OBSERVATIONDATETIME fell in DAD/SDS hospitalization (from ADMDATE to DDATE) or if OBSERVATIONDATETIME fell in ED visit (from REGTIME to DISPTIME).<br><br>Baseline eGFR was calculated from the closest value to index date in the year up to and including index date.<br>**eGFR slope (linear):**<br>During the extraction of baseline eGFR, the closest additional lab value by date that was at least 60 days earlier than the baseline value and within 2 years of the index date was similarly extracted.<br><br>eGFR slope at baseline was calculated as the change in eGFR divided by the number of years between the baseline and earlier values (a linear slope).
Dosage intensity during follow-up<br>Note: this variable is calculated by looking forward into the follow-up time included in the model	ODB	**This variable was created and used as a matching variable to force balance on dosage intensity between the HCTZ and CHLD groups.**<br><br>All HCTZ and CHLD prescriptions during follow-up where extracted with DAYSSUPL (prescription length in days) and QUANTITY information. STRNGTH (strength) was obtained from the DIN DRUGLIST data set. Total quantity of medication was calculated as QUANTITY multiplied by the cleaned strength variable. Total days supplied was calculated by summing the DAYSSUPL. Weighted average daily dose was calculated by dividing total quantity of medication by total days supplied.
Hypertension	DAD-SDS<br>NACRS<br><br>OHIP	**ICD10:** I10 I11 I12 I13 I15<br><br><br>**OHIP DX:** 401 402 403
Coronary Artery Disease (excluding angina)	DAD SDS<br>NACRS<br><br>OHIP	**ICD10:** I21 I22 Z955 T822<br>**CCI:** 1IJ50 1IJ76<br><br>**OHIP FEE:** R741 R742 R743 G298 E646 E651 E652 E654 E655 Z434 Z448<br>**OHIP DX:** 410 412
Myocardial Infarction	DAD SDS<br>NACRS	**ICD10:** I21, I22
Coronary Artery Bypass Grafting (CABG)	DAD SDS<br>NACRS<br><br>OHIP	**CCI:** 1IJ76<br><br><br>**OHIP FEE:** R742 R743 E654 E645 E652 E646
Congestive Heart Failure	DAD SDS	**ICD10:** I500 I501 I509 I255 J81

	NACRS<br><br><br>OHIP	**CCI:** 1HP53 1HP55 1HZ53GRFR 1HZ53LAFR 1HZ53SYFR<br><br>**OHIP FEE:** R701 R702 Z429<br>**OHIP DX:** 428
Atrial Fibrillation	DAD SDS<br>NACRS	**ICD10:** I48
Arrhythmia	DAD SDS<br>NACRS<br><br>OHIP	**ICD10:** I48 I44 I45 I47 I4900 I4901 I491 I492 I493 I494 I498 I499 R000 R001<br><br>**OHIP FEE:** G178 G179 G249 G261 G259 Z443 Z431 Z437
Ischemic Stroke	DAD SDS<br>NACRS	**ICD10:** H341 I630 I631 I632 I633 I634 I635 I638 I639 I64
Peripheral Vascular Disease	DAD SDS<br>NACRS<br><br><br><br>OHIP	**ICD 10:** I700 I702 I708 I709 I731 I738 I739 K551<br>**CCI:** 1KA76 1KA50 1KE76 1KG50 1KG57 1KG76MI 1KG87 1IA87LA 1IB87LA 1IC87LA 1ID87 1KA87LA 1KE57<br><br>**OHIP FEE:** R787 R780 R797 R804 R809 R875 R815 R936 R783 R784 R785 E626 R814 R786 R937 R860 R861 R855 R856 R933 R934 R791 E672 R794 R813 R867 E649
Diabetes	DAD SDS<br>NACRS<br><br>OHIP	**ICD10:** E10 E11 E13 E14<br><br><br>**OHIP FEE:** Q040 K029 K030 K045 K046<br>**OHIP DX:** 250
Chronic Obstructive Pulmonary Disorder	DAD SDS<br>NACRS	**ICD10:** J41 J43 J44
Chronic Liver Disease	DAD SDS<br>NACRS<br><br><br>OHIP	**ICD10:** B16 B17 B18 B19 I85 R17 R18 R160 R162 B942 Z225 E831 E830 K70 K713 K714 K715 K717 K721 K729 K73 K74 K753 K754 K758 K759 K76 K77<br><br>**OHIP FEE**: Z551 Z554<br>**OHIP DX :** 571 573 070
Major Cancer	DAD SDS<br>NACRS<br><br><br><br>OHIP	**ICD10:** 971 980 982 984 985 986 987 988 989 990 991 993 C15 C18 C19 C20 C22 C25 C34 C50 C56 C61 C82 C83 C85 C91 C92 C93 C94 C95 D00 D05 D010 D011 D012 D022 D075<br><br>**OHIP DX:** 203 204 205 206 207 208 150 154 155 157 162 174 175 183 185
Seizure	DAD SDS<br>NACRS	**ICD10:** G40 G41 R560 R568
Osteoporosis	DAD SDS<br>NACRS	**ICD10:** M80 M81 M82
Prior safety event – Syncope	DAD SDS<br>NACRS	**ICD10:** R55
Prior safety event – Fall	DAD SDS<br>NACRS	**ICD10:** W00 W01 W02 W03 W04 W05 W06 W07 W08 W09 W10 W11 W12 W13 W14 W15 W16 W17 W18 W19
Prior safety event – Fracture	DAD SDS<br>NACRS	**ICD10:** S720 S721 S52 S422 S723 S321 S322 S324 S323 S325 S327 S328 S825 S826 S827 S828 S829 S820 S821 S822 S823 S824 S222 S223 S224 S228 S229 S421 S420<br>**CCI:** 1VA73 1VC73 1VA74 1VA53 1VC74 1VA80 1TV73 1TV74 1TV03 1VC03 1VC80

© 2021 Edwards C et al. *JAMA Network Open.*

	OHIP	**OHIP:** F014 F022 F023 F025 F026 F028 F030 F032 F033 F046 F024 F027 F031 Z203 F095 F096 F097 Z211
Prior safety event - Hypotension	DAD SDS NACRS	**ICD10:** I95
Medications	ODB	

© 2021 Edwards C et al. *JAMA Network Open.*

eTable 3. List of Covariates Included in High Dimensional Propensity Score Matching Algorithm in Rank Order

Rank	Data source and code type	Code	Frequency type	Description
2	ODB drugname	NYSTATIN	once	NYSTATIN
2	OHIP FEE CODE	E078	once	MEDSPECASSESS&RE-ASSESS,COMPLEXMEDSPEC RE-ASSESS&PART ASSES
2	OHIP DX CODE	780	once	Convulsions, ataxia, vertigo, headache, except tension headache and migraine
5	ODB drugname	OMEPRAZOLE	sporadic	OMEPRAZOLE
5	OHIP FEE CODE	E083	sporadic	Subsequent visit by the MRP, to subsequent visit, C122, C123, C124, C142, C143, C882 or C982
5	OHIP DX CODE	787	sporadic	Anorexia, nausea and vomiting, heartburn, dysphagia, hiccough, hematemesis, jaundice, ascites, abdominal pain, melena, masses
8	ODB drugname	OMEPRAZOLE	once	OMEPRAZOLE
8	OHIP FEE CODE	E083	once	Subsequent visit by the MRP, to subsequent visit, C122, C123, C124, C142, C143, C882 or C982
8	OHIP DX CODE	787	once	Anorexia, nausea and vomiting, heartburn, dysphagia, hiccough, hematemesis, jaundice, ascites, abdominal pain, melena, masses
11	ODB drugname	OLMESARTAN MEDOXOMIL	once	OLMESARTAN MEDOXOMIL
11	OHIP FEE CODE	E082	once	Admission assessment by the MRP, to admission assessment
11	OHIP DX CODE	786	once	Epistaxis, hemoptysis, cough, dyspnea, masses, shortness of breath, hyperventilation, sleep apnea
14	ODB drugname	NYSTATIN	sporadic	NYSTATIN
14	OHIP FEE CODE	E078	sporadic	MEDSPECASSESS&RE-ASSESS,COMPLEXMEDSPEC RE-ASSESS&PART ASSES
14	OHIP DX CODE	780	sporadic	Convulsions, ataxia, vertigo, headache, except tension headache and migraine
18	ODB drugname	AZITHROMYCIN	once	AZITHROMYCIN
18	OHIP FEE CODE	A135	once	CONSULT.-INTERNAL MED.
18	DAD-SDS IDC10	K297	once	Gastritis, unspecified
18	NACRS ICD10	K922	once	Gastrointestinal haemorrhage, unspecified
18	DAD-SDS CCI	3IP10VY	once	Xray, heart with coronary arteries combined left and right heart catheterization (with or without fluoroscopy)
21	OHIP FEE CODE	G313	sporadic	D./.T.PROC CARDIOV ECG PROF.COMP-G.P.
23	ODB drugname	NYSTATIN	frequent	NYSTATIN

© 2021 Edwards C et al. *JAMA Network Open.*

23	OHIP FEE CODE	E078	frequent	MEDSPECASSESS&RE-ASSESS,COMPLEXMEDSPEC RE-ASSESS&PART ASSES
23	OHIP DX CODE	780	frequent	Convulsions, ataxia, vertigo, headache, except tension headache and migraine
25	ODB drugname	SPIRONOLACTONE	sporadic	SPIRONOLACTONE
26	OHIP FEE CODE	G313	frequent	D./.T.PROC CARDIOV ECG PROF.COMP-G.P.
27	ODB drugname	SPIRONOLACTONE	frequent	SPIRONOLACTONE
29	ODB drugname	OMEPRAZOLE	frequent	OMEPRAZOLE
29	OHIP FEE CODE	E083	frequent	Subsequent visit by the MRP, to subsequent visit, C122, C123, C124, C142, C143, C882 or C982
29	OHIP DX CODE	787	frequent	Anorexia, nausea and vomiting, heartburn, dysphagia, hiccough, hematemesis, jaundice, ascites, abdominal pain, melena, masses
33	ODB drugname	ACEBUTOLOL HCL	once	ACEBUTOLOL HCL
33	OHIP FEE CODE	A003	once	GEN. ASSESS. -F.P./G.P.
33	NACRS ICD10	A419	once	Sepsis, unspecified
33	DAD-SDS IDC10	C443	once	Malignant neoplasm skin of other and unspecified parts of face
33	DAD-SDS CCI	1CL89VRLM	once	Excision total, lens extracapsular phakoemulsification technique with insertion of folded posterior chamber lens prosthesis
36	OHIP FEE CODE	G578	once	CARD.DOPPLER&COMPL.1&2DIM ECHOCARDIOGRAPHY PRO.COMP.P1
37	OHIP FEE CODE	G571	once	ECHOCARDIOGRAPHY COMPL.STUDY 1&2DIM PROF.COMP.P1
38	OHIP FEE CODE	J128	once	DIAG. ULTRASOUND-ABDOMEN-ABDOMINAL SCAN - LIMITED STUDY.
39	ODB drugname	SPIRONOLACTONE	once	SPIRONOLACTONE
40	OHIP FEE CODE	J135	once	DIAG.ULTRASOUND ABD./RETROPERIT-ABDOMIN.SCAN COMPLETE
42	OHIP DX CODE	290	sporadic	Senile dementia, presenile dementia
42	NACRS ICD10	R64	sporadic	Cachexia
42	DAD-SDS IDC10	Z540	sporadic	Convalescence following surgery
44	OHIP FEE CODE	J202	once	DIAG.ULTRASOUND-DUPLEX SCAN SIMULT.REAL TIME.B MOD.PERIP.ART
46	ODB drugname	INACTIVATED INFLUENZA VIRUS	sporadic	INACTIVATED INFLUENZA VIRUS
46	OHIP FEE CODE	C132	sporadic	SUBSEQ. VISITS -UP TO 5 WKS. - INTERNAL MED. - HOSPITAL
46	OHIP DX CODE	560	sporadic	Intestinal obstruction, intussusception, paralytic ileus, volvulus, impaction of intestine
48	ODB drugname	ROSUVASTATIN CALCIUM	once	ROSUVASTATIN CALCIUM

49	OHIP FEE CODE	G313	once	D./.T.PROC CARDIOV ECG PROF.COMP-G.P.
51	OHIP DX CODE	290	once	Senile dementia, presenile dementia
51	NACRS ICD10	R64	once	Cachexia
51	DAD-SDS IDC10	Z540	once	Convalescence following surgery
53	OHIP FEE CODE	G310	frequent	D./.T.PROC.CARDIOV.ECG TECHNICAL COMP.
54	ODB drugname	DILTIAZEM HCL	once	DILTIAZEM HCL
55	OHIP FEE CODE	X410	once	DIAG. RADIOLOGY-COMPUTED TOMOGRAPHY-ABDOMEN-WITH I.V.CONTR.
56	OHIP FEE CODE	X091	frequent	DIAGNOSTIC RADIOLOGY CHEST 2 VIEWS
58	OHIP DX CODE	290	frequent	Senile dementia, presenile dementia
58	NACRS ICD10	R64	frequent	Cachexia
58	DAD-SDS IDC10	Z540	frequent	Convalescence following surgery
60	ODB drugname	NON PHARMACEUTICAL INGREDIENT	once	NON PHARMACEUTICAL INGREDIENT
61	ODB drugname	INSULIN ASPART RECOMBINANT	once	INSULIN ASPART RECOMBINANT
62	ODB drugname	PROCHLORPERAZINE MALEATE	once	PROCHLORPERAZINE MALEATE
63	ODB drugname	PERINDOPRIL TERT.BUTYLAMINE	frequent	PERINDOPRIL TERT.BUTYLAMINE
64	ODB drugname	NON PHARMACEUTICAL INGREDIENT	sporadic	NON PHARMACEUTICAL INGREDIENT
65	ODB drugname	WARFARIN SODIUM	frequent	WARFARIN SODIUM
66	ODB drugname	IRON FERROUS GLUCONATE	sporadic	IRON FERROUS GLUCONATE
67	OHIP FEE CODE	J202	sporadic	DIAG.ULTRASOUND-DUPLEX SCAN SIMULT.REAL TIME.B MOD.PERIP.ART
69	ODB drugname	OLMESARTAN MEDOXOMIL	frequent	OLMESARTAN MEDOXOMIL
69	OHIP FEE CODE	E082	frequent	Admission assessment by the MRP, to admission assessment
69	OHIP DX CODE	786	frequent	Epistaxis, hemoptysis, cough, dyspnea, masses, shortness of breath, hyperventilation, sleep apnea
71	ODB drugname	WARFARIN SODIUM	once	WARFARIN SODIUM
72	ODB drugname	WARFARIN SODIUM	sporadic	WARFARIN SODIUM
73	ODB drugname	NON PHARMACEUTICAL INGREDIENT	frequent	NON PHARMACEUTICAL INGREDIENT
74	OHIP DX CODE	427	once	Paroxysmal tachycardia, atrial or ventricular flutter or fibrillation, cardiac arrest, other arrythmias
75	ODB drugname	PANTOPRAZOLE SODIUM	frequent	PANTOPRAZOLE SODIUM
76	OHIP FEE CODE	X400	once	DIAG.RADIOLOGY-COMPUTED TOMOGRAHHY-HEAD-WITHOUT I.V.CONTRAST

© 2021 Edwards C et al. *JAMA Network Open.*

77	OHIP FEE CODE	H153	once	EMERG.DEPT.PHYS.ON DUTY SAT./SUN/HOLIDAY MULT.SYST.ASSESS.
78	ODB drugname	DIGOXIN	sporadic	DIGOXIN
79	ODB drugname	DILTIAZEM HCL	frequent	DILTIAZEM HCL
80	OHIP FEE CODE	J128	sporadic	DIAG. ULTRASOUND-ABDOMEN-ABDOMINAL SCAN - LIMITED STUDY.
82	ODB drugname	INACTIVATED INFLUENZA VIRUS	frequent	INACTIVATED INFLUENZA VIRUS
82	OHIP FEE CODE	C132	frequent	SUBSEQ. VISITS -UP TO 5 WKS. - INTERNAL MED. - HOSPITAL
82	OHIP DX CODE	560	frequent	Intestinal obstruction, intussusception, paralytic ileus, volvulus, impaction of intestine
84	ODB drugname	DILTIAZEM HCL	sporadic	DILTIAZEM HCL
85	OHIP FEE CODE	A605	once	CONSULT.-CARDIOLOGY
86	OHIP FEE CODE	E017	once	PATIENTS ASA 4 - PATIENT WITH INCAPACITATING
87	ODB drugname	INSULIN ASPART RECOMBINANT	sporadic	INSULIN ASPART RECOMBINANT
88	OHIP FEE CODE	G315	once	CARDIOV.MAX.STRESS E.C.G. TECH.COMP.
89	OHIP FEE CODE	X185	sporadic	DIAGNOSTIC RADIOLOGY-MAMMOGRAM-BILATERAL
90	OHIP FEE CODE	X232	once	DIAG.RAD.-PELVIS WITH I.V. CONTRAST
91	ODB drugname	PERINDOPRIL TERT.BUTYLAMINE	sporadic	PERINDOPRIL TERT.BUTYLAMINE
92	OHIP FEE CODE	G391	once	D./T. PROC-OTHER RESUSCITATION-AFT 1ST 1/4HR.(PER 1/4HR).
93	ODB drugname	PANTOPRAZOLE SODIUM	sporadic	PANTOPRAZOLE SODIUM
94	OHIP FEE CODE	X185	once	DIAGNOSTIC RADIOLOGY-MAMMOGRAM-BILATERAL
95	ODB drugname	TERAZOSIN HCL	once	TERAZOSIN HCL
96	OHIP DX CODE	401	once	Essential, benign hypertension
97	ODB drugname	IRON FERROUS GLUCONATE	frequent	IRON FERROUS GLUCONATE
98	OHIP FEE CODE	G319	once	CARDIOV.MAX.STRESS E.C.G. PROF COMP.
99	ODB drugname	PANTOPRAZOLE SODIUM	once	PANTOPRAZOLE SODIUM
100	ODB drugname	PROCHLORPERAZINE MALEATE	frequent	PROCHLORPERAZINE MALEATE
101	OHIP FEE CODE	H152	once	SAT/SUN & HOLIDAYS COMPREHENSIVE ASSESS. & CARE
102	OHIP FEE CODE	J135	sporadic	DIAG.ULTRASOUND ABD./RETROPERIT-ABDOMIN.SCAN COMPLETE
104	ODB drugname	INACTIVATED INFLUENZA VIRUS	once	INACTIVATED INFLUENZA VIRUS

© 2021 Edwards C et al. *JAMA Network Open.*

104	OHIP FEE CODE	C132	once	SUBSEQ. VISITS -UP TO 5 WKS. - INTERNAL MED. - HOSPITAL
104	OHIP DX CODE	560	once	Intestinal obstruction, intussusception, paralytic ileus, volvulus, impaction of intestine
108	ODB drugname	AMILORIDE HCL	once	AMILORIDE HCL
108	OHIP DX CODE	162	once	Bronchus, lung
108	DAD-SDS IDC10	H269	once	Cataract, unspecified
108	NACRS ICD10	I500	once	Congestive heart failure
108	DAD-SDS CCI	1VG53LAPPN	once	Implantation of internal device, knee joint with synthetic material
111	ODB drugname	INSULIN LISPRO RECOMBINANT	once	INSULIN LISPRO RECOMBINANT
112	OHIP DX CODE	486	frequent	Pneumonia - all types
113	OHIP FEE CODE	X407	once	DIAG. RADIOLOGY-COMPUTED TOMOGRAPHY-THORAX-WITH I.V.CONTR.
114	OHIP FEE CODE	J163	once	PELVIS, LIMITED STUDY OTHER THAN PREGNANCY
115	OHIP DX CODE	799	sporadic	Other ill-defined conditions
116	OHIP FEE CODE	G271	once	D./T. PROC.-CARDIOV.-ANTICOAGULANT SUPERVISION
117	OHIP DX CODE	402	once	Hypertensive heart disease
118	ODB drugname	TIOTROPIUM BROMIDE	frequent	TIOTROPIUM BROMIDE
119	ODB drugname	GLICLAZIDE	frequent	GLICLAZIDE
120	OHIP DX CODE	959	frequent	Other injuries or trauma
123	ODB drugname	AMILORIDE HCL	sporadic	AMILORIDE HCL
123	OHIP DX CODE	162	sporadic	Bronchus, lung
123	DAD-SDS IDC10	H269	sporadic	Cataract, unspecified
123	NACRS ICD10	I500	sporadic	Congestive heart failure
123	DAD-SDS CCI	1VG53LAPPN	sporadic	Implantation of internal device, knee joint with synthetic material
126	OHIP FEE CODE	Q150	once	FOBT DISTRIBUTION AND COUNSELLING FEE
128	OHIP DX CODE	289	once	Other diseases of blood, marrow, spleen
128	NACRS ICD10	R600	once	Localized oedema
128	DAD-SDS IDC10	Z538	once	Procedure not carried out for other reasons
130	ODB drugname	DIGOXIN	once	DIGOXIN
131	ODB drugname	IRON FERROUS GLUCONATE	once	IRON FERROUS GLUCONATE
132	OHIP DX CODE	427	sporadic	Paroxysmal tachycardia, atrial or ventricular flutter or fibrillation, cardiac arrest, other arrythmias
133	ODB drugname	MELOXICAM	once	MELOXICAM
134	ODB drugname	METOPROLOL TARTRATE	once	METOPROLOL TARTRATE
135	ODB drugname	CYANOCOBALAMIN	once	CYANOCOBALAMIN
136	OHIP FEE CODE	J200	once	DIAG.ULTRA-VASC.SYST-ANK.PRESS.MEA+SEG.PRES/PULS.VOL/DOPPLER

© 2021 Edwards C et al. *JAMA Network Open.*

137	OHIP DX CODE	486	sporadic	Pneumonia - all types
138	OHIP DX CODE	402	sporadic	Hypertensive heart disease
139	ODB drugname	TERAZOSIN HCL	sporadic	TERAZOSIN HCL
140	OHIP DX CODE	682	once	Cellulitis, abscess
141	OHIP FEE CODE	J323	once	PUL.FUNCT.O2 SATURATION BY OXIMETRY
143	OHIP DX CODE	280	sporadic	Iron deficiency anaemia
143	NACRS ICD10	R33	sporadic	Retention of urine
143	DAD-SDS IDC10	Z031	sporadic	Observation for suspected malignant neoplasm
145	ODB drugname	INSULIN ASPART RECOMBINANT	frequent	INSULIN ASPART RECOMBINANT
146	OHIP FEE CODE	G395	once	D./T. PROC-OTHER RESUSCITATION-1ST 1/4HR. PER PHYS.
147	ODB drugname	PERINDOPRIL TERT.BUTYLAMINE	once	PERINDOPRIL TERT.BUTYLAMINE
149	OHIP DX CODE	280	once	Iron deficiency anaemia
149	NACRS ICD10	R33	once	Retention of urine
149	DAD-SDS IDC10	Z031	once	Observation for suspected malignant neoplasm
151	ODB drugname	NITROFURANTOIN	once	NITROFURANTOIN
152	OHIP FEE CODE	G401	once	CRIT.INTENS.CARE EXCL.VENTIL.SUP-PHYS.IN CHGE.2ND-10TH DAY
153	OHIP FEE CODE	J200	sporadic	DIAG.ULTRA-VASC.SYST-ANK.PRESS.MEA+SEG.PRES/PULS.VOL/DOPPLER
154	ODB drugname	TELMISARTAN	once	TELMISARTAN
155	OHIP DX CODE	959	once	Other injuries or trauma
156	ODB drugname	HUMAN INSULIN ISOPHANE RECOMBINANT	once	HUMAN INSULIN ISOPHANE RECOMBINANT
157	OHIP FEE CODE	J201	once	DIAG.ULTRASOUND-DUPLEX SCAN SIMULT.REAL TIME.B MODE VASC.SYS
158	OHIP FEE CODE	J850	once	NUCLEAR MED.CORRES.TO J650
159	OHIP FEE CODE	H102	once	COMPREHENSIVE ASSESS. & CARE
160	ODB drugname	VALSARTAN	once	VALSARTAN
161	OHIP FEE CODE	X406	once	DIAG. RADIOLOGY-COMPUTED TOMOGRAPHY-THORAX-WITHOUT I.V.CONTR
162	OHIP FEE CODE	G310	once	D./.T.PROC.CARDIOV.ECG TECHNICAL COMP.
163	OHIP FEE CODE	H154	once	EMERG.DEPT.PHYS.ON DUTY SAT./SUN./HOLIDAY REASSESS.
164	OHIP DX CODE	821	once	Femur
165	OHIP FEE CODE	Q131	once	MAMMOGRAM TRACKING CODE
166	ODB drugname	METOPROLOL	once	METOPROLOL
167	OHIP FEE CODE	H134	once	GP-REASSESS-EMERG DEPT-PHYSICIAN ON DUTY M-F EVENINGS

© 2021 Edwards C et al. *JAMA Network Open.*

168	ODB drugname	SENNA	frequent	SENNA
169	OHIP FEE CODE	K030	sporadic	DIABETIC MANAGEMENT FEE
170	OHIP FEE CODE	Q133	once	COLORECTAL SCREENING TRACKING CODE
171	OHIP FEE CODE	X409	once	DIAG. RADIOLOGY-COMPUTED TOMOGRAPHY-ABDOMEN-W'OUT I.V.CONTR.
172	ODB drugname	DIGOXIN	frequent	DIGOXIN
173	OHIP FEE CODE	J327	once	PULM.FUNC.-REPEAT J304 AFTER BRONCHODILATOR
174	OHIP FEE CODE	X487	once	WHEN GADOLINIUM IS USED, ... ADD
175	OHIP FEE CODE	G372	once	D./T. PROC.-INJECTIONS-INTRADERMAL/MUSCULAR ETC. EA. ADD.
176	OHIP FEE CODE	G271	sporadic	D./T. PROC.-CARDIOV.-ANTICOAGULANT SUPERVISION
177	OHIP FEE CODE	K030	once	DIABETIC MANAGEMENT FEE
178	ODB drugname	GLICLAZIDE	once	GLICLAZIDE
179	OHIP DX CODE	459	once	Other disorders of circulatory system
180	OHIP FEE CODE	G401	sporadic	CRIT.INTENS.CARE EXCL.VENTIL.SUP-PHYS.IN CHGE.2ND-10TH DAY
181	ODB drugname	ROSUVASTATIN CALCIUM	sporadic	ROSUVASTATIN CALCIUM
182	OHIP FEE CODE	X155	sporadic	BONE MINERAL DENSITY HIGH RISK 2+SITES
183	OHIP FEE CODE	H105	once	INTERIM INPATIENT ADMISSION ORDERS
184	OHIP DX CODE	627	once	Menopause, post-menopausal bleeding
185	OHIP DX CODE	401	frequent	Essential, benign hypertension
186	ODB drugname	LORAZEPAM	once	LORAZEPAM
187	OHIP DX CODE	821	sporadic	Femur
188	ODB drugname	TERAZOSIN HCL	frequent	TERAZOSIN HCL
189	OHIP FEE CODE	X155	once	BONE MINERAL DENSITY HIGH RISK 2+SITES
191	ODB drugname	CLOPIDOGREL BISULFATE	frequent	CLOPIDOGREL BISULFATE
191	OHIP DX CODE	332	frequent	Parkinson's disease
191	NACRS ICD10	Z098	frequent	Follow-up examination after other treatment for other conditions
193	OHIP FEE CODE	J327	sporadic	PULM.FUNC.-REPEAT J304 AFTER BRONCHODILATOR
194	ODB drugname	VENLAFAXINE HCL	frequent	VENLAFAXINE HCL
195	ODB drugname	SENNA	sporadic	SENNA
196	OHIP FEE CODE	J310	once	PULM/FUNC-SINGLE BREATH DIFFUSING CAPACITY.
197	OHIP FEE CODE	J304	frequent	PULM/FUNC. FLOW VOL.LOOP-STANDARD LUNG MECHANICS
198	ODB drugname	LORAZEPAM	sporadic	LORAZEPAM
200	ODB drugname	CITALOPRAM HBR	sporadic	CITALOPRAM HBR

© 2021 Edwards C et al. *JAMA Network Open.*

200	OHIP DX CODE	300	sporadic	Anxiety neurosis, hysteria, neurasthenia, obsessive compulsive neurosis, reactive depression
200	NACRS ICD10	S22300	sporadic	Fracture of rib, closed

© 2021 Edwards C et al. *JAMA Network Open.*

eTable 4. Mean Follow-up Time Among Chlorthalidone and Hydrochlorothiazide Users Overall and by eGFR Category

FOLLOW-UP TIMES Mean (SD) Days	CHLORTHALIDONE					HYDROCHLOROTHIAZIDE			
	Overall Population	eGFR ≥60 mL/min/1.73m^2	eGFR 45-59 mL/min/1.73m^2	eGFR <45 mL/min/1.73m^2		Overall Population	eGFR ≥60 mL/min/1.73m^2	eGFR 45-59 mL/min/1.73m^2	eGFR <45 mL/min/1.73m^2
**≥30% eGFR Decline**	658 (338)	678 (335)	620 (333)	604 (350)		741 (294)	759 (284)	700 (312)	661 (321)
**Dialysis/Kidney Transplant**	812 (331)	820 (327)	805 (334)	778 (343)		896 (258)	905 (250)	878 (272)	850 (296)
**Cardiovascular Event**	652 (398)	667 (397)	645 (398)	585 (400)		742 (369)	761 (361)	696 (379)	669 (397)
**All-Cause Mortality**	815 (329)	821 (326)	808 (334)	791 (337)		897 (257)	906 (249)	879 (272)	861 (290)
**Hypokalemia**	685 (395)	682 (394)	698 (396)	682 (398)		831 (321)	840 (316)	811 (333)	791 (342)
**Hyperkalemia**	814 (331)	826 (326)	804 (334)	770 (345)		900 (258)	914 (244)	875 (277)	832 (313)
**Hyponatremia**	781 (356)	786 (354)	775 (358)	759 (362)		869 (293)	880 (286)	841 (313)	837 (313)

© 2021 Edwards C et al. *JAMA Network Open.*

eTable 5. Sensitivity Analyses of Associations of Chlorthalidone vs Hydrochlorothiazide With Study Outcomes

**HAZARD RATIOS (95% CI) Chlorthalidone vs. Hydrochlorothiazide**	**Overall Population**	**eGFR ≥60 mL/min/1.73m^2**	**eGFR 45-59 mL/min/1.73m^2**	**eGFR <45 mL/min/1.73m^2**	**P-Value for Interaction**
**≥30% eGFR DECLINE**					
**Model 1:** 1°Model ('intention to treat', 1mg:2mg dose-matching)	1.24 (1.13-1.36)	1.28 (1.13-1.44)	1.37 (1.13-1.67)	0.98 (0.78-1.24)	0.08
**Model 2:** 'Intention to treat', 1mg:3mg dose-matching	1.30 (1.18-1.43)	1.25 (1.11-1.42)	1.55 (1.27-1.89)	1.08 (0.84-1.39)	0.07
**Model 3:** 'As treated', 1mg:2mg dose-matching	1.31 (1.11-1.54)	1.47 (1.21-1.80)	1.25 (0.90-1.73)	0.85 (0.55-1.30)	0.07
**Model 4:** Matching on antihypertensive medication use	1.37 (1.07-1.76)	1.36 (1.02-1.80)	1.69 (0.94-3.04)	0.85 (0.29-2.44)	0.53
**Model 5:** Thiazide monotherapy[1]	1.55 (0.79-3.05)	--	--	--	--
**DIALYSIS OR KIDNEY TRANSPLANTATION**					
**Model 1:** 1°Model ('intention to treat', 1mg:2mg dose-matching)	1.44 (0.88-2.36)	2.18 (0.97-4.91)	1.61 (0.58-4.49)	1.11 (0.57-2.15)	0.43
**Model 2:** 'Intention to treat', 1mg:3mg dose-matching	2.01 (1.17-3.44)	2.20 (0.92-5.24)	3.33 (0.98-11.4)	1.43 (0.70-2.92)	0.43
**Model 3:** 'As treated', 1mg:2mg dose-matching	1.44 (0.66-3.11)	2.07 (0.62-6.91)	1.05 (0.21-5.17)	1.25 (0.38-4.10)	0.76
**Model 4:** Matching on antihypertensive medication use[2]	--	--	--	--	--
**Model 5:** Thiazide monotherapy[1,2]	--	--	--	--	--
**CARDIOVASCULAR EVENT**					
**Model 1:** 1°Model ('intention to treat', 1mg:2mg dose-matching)	1.12 (1.04-1.22)	1.20 (1.09-1.32)	0.97 (0.81-1.16)	1.03 (0.84-1.27)	0.08
**Model 2:** 'Intention to treat', 1mg:3mg dose-matching	1.15 (1.06-1.25)	1.22 (1.10-1.35)	0.99 (0.82-1.19)	1.02 (0.82-1.28)	0.08
**Model 3:** 'As treated', 1mg:2mg dose-matching	1.13 (1.01-1.26)	1.19 (1.04-1.36)	0.89 (0.69-1.13)	1.21 (0.93-1.58)	0.10
**Model 4:** Matching on antihypertensive medication use	1.12 (0.85-1.47)	1.12 (0.83-1.49)	1.29 (0.65-2.58)	0.80 (0.24-2.70)	0.79
**Model 5:** Thiazide monotherapy[1]	1.14 (0.63-2.05)	--	--	--	--
**ALL-CAUSE MORTALITY**					
**Model 1:** 1°Model ('intention to treat', 1mg:2mg dose-matching)	1.10 (0.93-1.29)	1.27 (1.02-1.58)	1.21 (0.87-1.68)	0.73 (0.52-1.03)	0.02
**Model 2:** 'Intention to treat', 1mg:3mg dose-matching	1.16 (0.98-1.37)	1.30 (1.04-1.64)	1.18 (0.83-1.67)	0.78 (0.54-1.11)	0.05
**Model 3:** 'As treated', 1mg:2mg dose-matching	1.26 (0.89-1.78)	1.32 (0.82-2.11)	2.04 (1.09-3.81)	0.57 (0.25-1.32)	0.06
**Model 4:** Matching on antihypertensive medication use	0.86 (0.54-1.39)	0.83 (0.48-1.46)	1.06 (0.42-2.70)	0.78 (0.16-3.79)	0.89
**Model 5:** Thiazide monotherapy[1]	1.16 (0.43-3.17)	--	--	--	--
**HYPOKALEMIA**					
**Model 1:** 1°Model ('intention to treat', 1mg:2mg dose-matching)	1.70 (1.55-1.87)	1.86 (1.67-2.08)	1.57 (1.25-1.96)	1.10 (0.84-1.45)	0.001

© 2021 Edwards C et al. *JAMA Network Open.*

**Model 2:** ‘Intention to treat’, 1mg:3mg dose-matching	1.77 (1.60-1.95)	1.92 (1.72-2.15)	1.62 (1.28-2.04)	1.15 (0.86-1.55)	0.005
**Model 3:** ‘As treated’, 1mg:2mg dose-matching	2.43 (2.15-2.74)	2.54 (2.21-2.92)	2.31 (1.72-3.10)	1.93 (1.33-2.80)	0.36
**Model 4:** Matching on antihypertensive medication use	1.78 (1.39-2.27)	1.89 (1.45-2.45)	1.15 (0.59-2.23)	1.81 (0.53-6.16)	0.38
**Model 5:** Thiazide monotherapy[1]	1.62 (1.09-2.40)	--	--	--	--
**HYPERKALEMIA**					
**Model 1:** 1°Model (‘intention to treat’, 1mg:2mg dose-matching)	1.05 (0.79-1.39)	1.21 (0.77-1.92)	1.13 (0.68-1.89)	0.86 (0.54-1.37)	0.55
**Model 2:** ‘Intention to treat’, 1mg:3mg dose-matching	1.33 (0.99-1.77)	1.28 (0.79-2.05)	1.30 (0.77-2.21)	1.24 (0.75-2.07)	0.99
**Model 3:** ‘As treated’, 1mg:2mg dose-matching	1.35 (0.87-2.08)	1.53 (0.76-3.07)	1.84 (0.85-3.97)	0.93 (0.46-1.91)	0.41
**Model 4:** Matching on antihypertensive medication use[2]	--	--	--	--	--
**Model 5:** Thiazide monotherapy[1,2]	--	--	--	--	--
**HYPONATREMIA**					
**Model 1:** 1°Model (‘intention to treat’, 1mg:2mg dose-matching)	1.14 (0.98-1.32)	1.14 (0.95-1.36)	1.06 (0.75-1.49)	1.24 (0.82-1.85)	0.84
**Model 2:** ‘Intention to treat’, 1mg:3mg dose-matching	1.13 (0.97-1.32)	1.12 (0.93-1.35)	1.12 (0.79-1.60)	1.19 (0.76-1.86)	0.97
**Model 3:** ‘As treated’, 1mg:2mg dose-matching	1.35 (1.11-1.64)	1.35 (1.07-1.71)	1.34 (0.87-2.06)	1.31 (0.71-2.43)	0.99
**Model 4:** Matching on antihypertensive medication use	1.06 (0.71-1.57)	1.23 (0.79-1.92)	0.72 (0.31-1.66)	0.34 (0.03-3.72)	0.33
**Model 5:** Thiazide monotherapy[1]	1.28 (0.54-3.03)	--	--	--	--

[1] Unable to stratify by eGFR category for the monotherapy model as total N = 733 with 91% (669/733) in the eGFR ≥60 mL/min/1.73m^2 category.

[2] Not feasible due to too few events.

© 2021 Edwards C et al. *JAMA Network Open.*

eReferences

1. Levy AR, O'Brien BJ, Sellors C, Grootendorst P, Willison D. Coding accuracy of administrative drug claims in the Ontario Drug Benefit database. *Can J Clin Pharmacol.* 2003;10(2):67-71.
2. Statistics Canada. Table 17-10-0022-01 Estimates of interprovincial migrants by province or territory of origin and destination, annual. Accessed April 29, 2019.

© 2021 Edwards C et al. *JAMA Network Open.*
